# Computational Identification of Dithymoquinone as a Potential Inhibitor of Myostatin and Regulator of Muscle Mass

**DOI:** 10.3390/molecules26175407

**Published:** 2021-09-06

**Authors:** Syed Sayeed Ahmad, Khurshid Ahmad, Eun Ju Lee, Sibhghatulla Shaikh, Inho Choi

**Affiliations:** 1 Department of Medical Biotechnology, Yeungnam University, Gyeongsan 38541, Korea; sayeedahmad4@gmail.com (S.S.A.); ahmadkhursheed2008@gmail.com (K.A.); gorapadoc0315@hanmail.net (E.J.L.); sibhghat.88@gmail.com (S.S.); 2 Research Institute of Cell Culture, Yeungnam University, Gyeongsan 38541, Korea

**Keywords:** myostatin, dithymoquinone, natural compounds, molecular dynamics, ActR2B, protein–protein interaction

## Abstract

The skeletal muscle (SM) is the largest organ in the body and has tremendous regenerative power due to its myogenic stem cell population. Myostatin (MSTN), a protein produced by SM, is released into the bloodstream and is responsible for age-related reduced muscle fiber development. The objective of this study was to identify the natural compounds that inhibit MSTN with therapeutic potential for the management of age-related disorders, specifically muscle atrophy and sarcopenia. Sequential screening of 2000 natural compounds was performed, and dithymoquinone (DTQ) was found to inhibit MSTN with a binding free energy of −7.40 kcal/mol. Furthermore, the docking results showed that DTQ reduced the binding interaction between MSTN and its receptor, activin receptor type-2B (ActR2B). The global energy of MSTN-ActR2B was found to be reduced from −47.75 to −40.45 by DTQ. The stability of the DTQ–MSTN complex was subjected to a molecular dynamics analysis for up to 100 ns to check the stability of the complex using RMSD, RMSF, Rg, SASA, and H-bond number. The complex was found to be stable after 10 ns to the end of the simulation. These results suggest that DTQ blocks MSTN signaling through ActR2B and that it has potential use as a muscle growth-promoting agent during the aging process.

## 1. Introduction

Human skeletal muscle (SM) is a highly plastic tissue that accounts for up to 40% of total body weight and 50–75% of body protein [1]. SM is the largest body organ and is mainly responsible for movement, temperature control, and maintaining glucose levels because muscle contraction utilizes glucose as a fuel source [2]. Furthermore, SM has considerable regenerative potential in response to damage or disease due to its myogenic stem cell population [3]. The maintenance of SM mass depends on the balance between protein synthesis and degradation, which are highly sensitive to hormonal balance, nutritional status, exercise, injury, and disease [4]. Loss of SM mass is a marker of several pathologies, such as diabetes, obesity, cancer, and aging [4], and it has been studied extensively by our group [5,6,7,8,9]. Aging is a difficult issue to address and has become a priority due to rapid increases in elderly populations and age-related diseases [10].

The progressive loss of SM mass during aging is termed sarcopenia, which is described as a decline of muscle quality and quantity [11]. Myostatin (MSTN) is a protein secreted by myocytes and is reportedly a negative regulator of SM mass and growth. MSTN is expressed during embryogenesis by cells in developing SM and acts to regulate muscle fiber numbers. During aging, MSTN is released by SM to blood and limits muscle fiber growth. The active form of MSTN binds to its receptor, activin receptor type-2B (ActR2B), and thus activates signaling for protein degradation via Smad2/3-mediated transcription. Furthermore, by blocking Akt signaling, Smad activation inhibits muscle protein synthesis. In various disease models, including cancer-associated cachexia, pharmacological blockade of the MSTN/activin- ActR2B pathway has been shown to prevent loss of muscle mass and strength [12,13,14]. Numerous biopharmaceutical agents that inactivate MSTN binding are being tested in clinical trials as potential treatments for muscle-wasting diseases and muscular dystrophies [15]. The importance of MSTN has been reported in several disease conditions, including cachexia, sarcopenia, muscular atrophy, and other muscular dystrophies such as Duchenne muscular dystrophy, and its inhibition is an important strategy for managing these disease conditions [16,17,18,19,20]. Muscle loss happens as a result of various chronic illnesses (cachexia) as well as natural aging (sarcopenia). Sarcopenia, or the age-related decrease in muscle mass and function, is a prevalent disease in older persons and is linked to a number of negative health consequences. Various negative regulators (MSTN, atrogin-1, muscle ring finger-1, nuclear factor-kappaB (NF-B)) have been proposed to promote protein degradation during both sarcopenia and cachexia [21,22,23]. The potential of MSTN inhibition is efficient to work as an anti-sarcopenia and anti-cachexia agent. For these reasons, research is being conducted on the design of new MSTN inhibitors that promote muscle regeneration after injury [24].

Natural products and their molecular frameworks are valuable resources for drug discovery and design [25], and molecular interaction studies provide a means of identifying drug-like molecules [26,27,28,29]. Studies on the pharmacological properties (such as antidiabetic, anticancer, immunomodulator, and analgesic properties) of natural compounds are being actively pursued [30,31]. In the present study, we sought to identify the natural inhibitors of MSTN, with a view toward finding a novel means of managing age-related disorders and treating muscle atrophy and sarcopenia.

## 2. Results

A total of 1500 of a prepared library of 2000 satisfied Lipinski’s rule of five (RO5) and were subjected to a structure-based virtual screening against the active site of MSTN. Compounds with a binding free energy of better than −6.0 kcal/mol (20 compounds shown in Table S1) were considered for further analysis. Finally, the four top compounds were selected based on their drug-likeness properties. Of these four compounds, dithymoquinone (DTQ) had the better binding free energy (−7.40 kcal/mol). Based on its better binding free energy, RO5, and Swiss ADME (Table 1), DTQ was studied in depth using molecular docking and dynamics simulation analysis.

The ADME properties of the four top compounds are listed in Table 2.

DTQ was predicted to be able to cross the blood–brain barrier and to be absorbed in the gastrointestinal tract, as shown in Supplementary Figure S1B, which was drawn between the WLOGP (logP method developed by Wildman and Crippen) and TPSA (topological polar surface area). DTQ is represented by a red dot in the ellipse and following the bioavailability radar, which was used for the rapid appraisal of drug-likeness as shown in Supplementary Figure S1A. Subsequently, DTQ was checked for cardiotoxicity and found to be non-cardiotoxic (Supplementary Figure S1C).

Free energy of binding, obtained by Autodock, for complexes between MSTN and the four selected compounds DTQ, calycosin, limonin, and nigellidine were −7.40, −6.60, −6.85, and −6.82 kcal/mol, respectively. To confirm these results, multiple scoring docking was performed to check interactions and binding free energies. The free energies of binding obtained using multiple docking tools are provided in Table 3.

**Figure 1:**
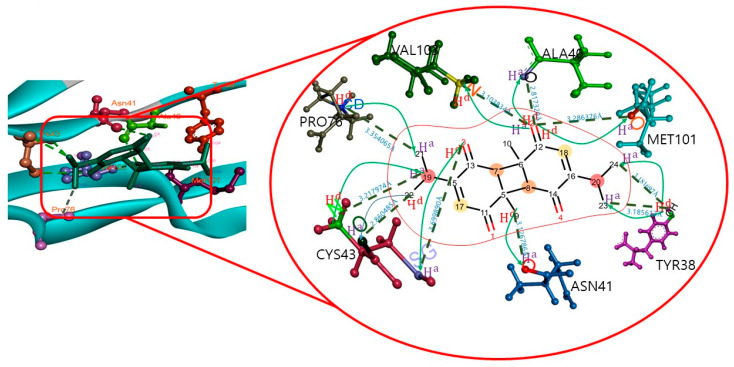
Atomic level interaction between MSTN and DTQ determined by docking.

The docking results showed that DTQ interacted with different amino acid residues of the MSTN chain A, that is, LEU20, VAL22, TYR38, ALA40, ASN41, TYR42, CYS43, PRO76, MET101, VAL102, and VAL103. DTQ formed the H-bonds with MSTN (Table 4) TYR38:OH—DTQ:O24, TYR38:OH—DTQ:O23, CYS43:N—DTQ:O19, VAL103:N—DTQ:O2, DTQ: O2—ALA40:O, DTQ:O2—MET101:O, DTQ:O3—CYS43:SG, DTQ:O9—ASN41:O, DTQ:O22—CYS43:O, and PRO76:CD—DTQ:O21, which had H-bond distances of 3.18, 3.18, 3.21, 3.10, 2.81, 3.28, 3.69, 3.12, 2.86, and 3.35 Å, respectively (Figure 1).

**Figure 2:**
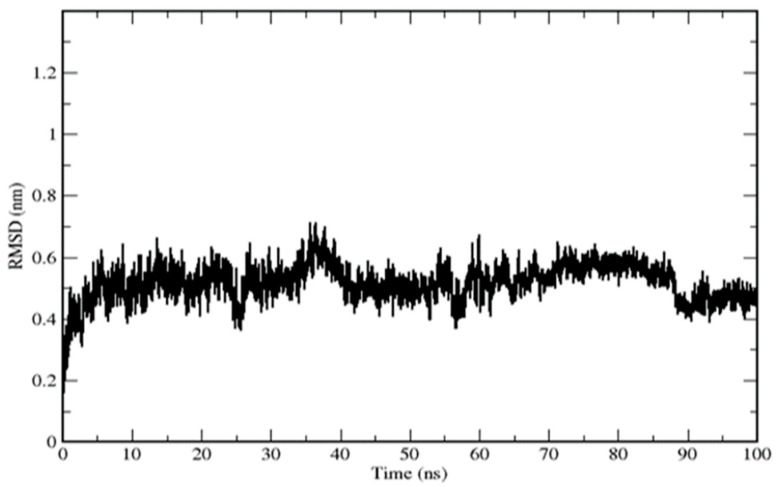
Root-mean-square deviations of the MSTN–DTQ complex.

Furthermore, residues LEU20, VAL22, TYR42, and VAL102 were involved in hydrophobic interactions. In this complex, DTQ was shown to interact with the different amino acids of the target along with their H-bond distances. The DTQ–MSTN complex was subjected to molecular dynamics analysis study for up to 100ns, and RMSD, RMSF, Rg, SASA, and number of H-bonds were analyzed. The complex exhibited deviations during the initial 10 ns, and an RMSD of ~0.18–0.60 nm was found throughout the simulations (Figure 2).

**Figure 3:**
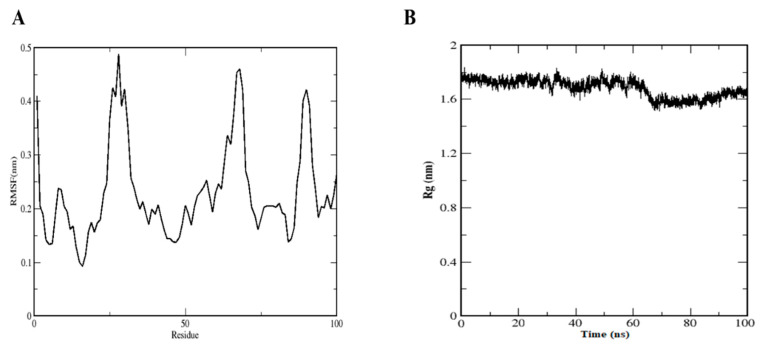
(**A**) Root-mean-square fluctuation of the DTQ–MSTN complex. (**B**) Radius of gyration of DTQ–MSTN complex.

The RMSF diagram showed fluctuations at N and C terminals of MSTN. Backbone residue fluctuation ranged between 0.1 and 0.5 nm. In the case of Rg, during the initial stage, it was higher at 1.80 nm but diminished to 1.60 nm at 8.0 ns and then remained stable throughout the remainder of the simulation (Figure 3A).

**Figure 4:**
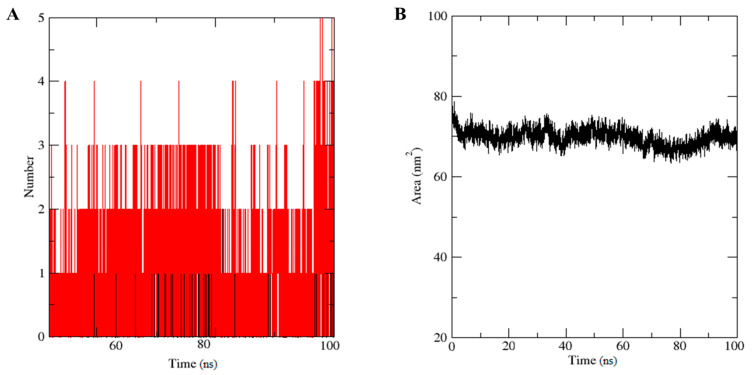
(**A**) Number of H-bonds in the DTQ–MSTN complex. (**B**) Solvent accessible surface area (SASA) of the DTQ–MSTN complex.

H-bond number is directly related to complex stability (Figure 4A). Five H-bonds were formed during the simulation time, though two H-bonds were present constantly throughout the simulation. Solvent accessible surface area (SASA) is used to determine the solvent accessibility of proteins. The SASA graph showed the same pattern as the gyration radius (Figure 3B), which was initially large (78 nm^2^) (Figure 4B).

**Figure 5:**
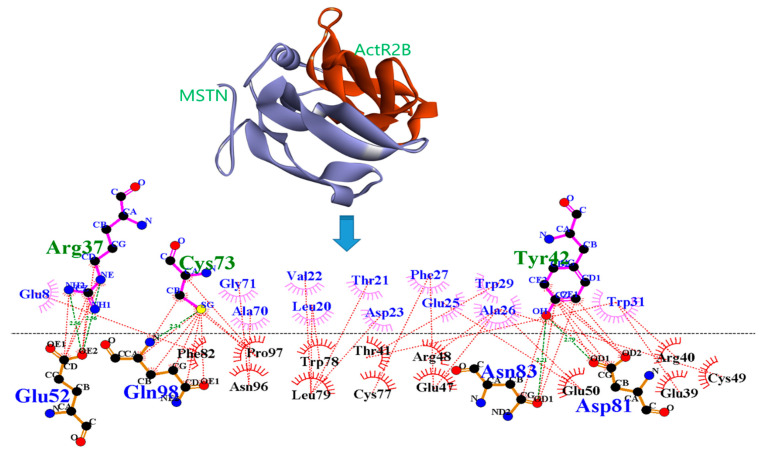
PPI diagram for MSTN and ActR2B. Green dotted lines indicate an H-Bond and brown dotted lines indicate hydrophobic interactions.

Protein–protein interaction (PPI) analysis [32] was used to investigate the interaction between MSTN and ActR2B (Figure 5).

**Figure 6:**
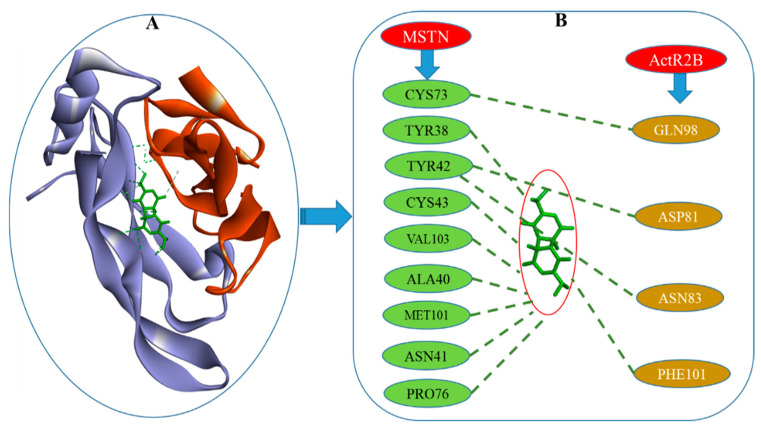
PPI diagram: (**A**) The structure of the MSTN–DTQ–ActR2B complex. (**B**) Amino acid residues that interact in MSTN–DTQ–ActR2B.

MSTN–DTQ was further docked with ActR2B to check the effect of the MSTN–DTQ–ActR2B complex formation on MSTN and ActR2B binding, which was found to be reduced. The free energy of binding was found to be −7.40 kcal/mol for MSTN–DTQ, and when the MSTN–DTQ complex was docked with ActR2B, FireDock showed a reduction in global energy from −47.75 to −40.45 (Figure 6).

## 3. Discussion

Virtual screening is useful for identifying drug-like compounds [33] and for checking their affinities with desired therapeutic targets [34]. In the present study, we found in silico that DTQ potently inhibited MSTN and disrupted MSTN–ActR2B interaction, which suggests that DTQ is a potential MSTN inhibitor with muscle growth-promoting effects [35]. MSTN–ActR2B complex interruption has been reported to be an effective strategy for treating SM-related disorders [13], and inhibition of MSTN/activin activity was reported to recover insulin sensitivity, reduce unnecessary adiposity, attenuate systemic inflammation, and accelerate bone fracture healing [13]. DTQ is found naturally in *Nigella sativa*, which is used in traditional medicine. The seeds of this plant are used as a nutritional flavoring agent and as a remedy for many ailments, and its components have been reported to have immune stimulatory, anti-inflammatory, hypoglycemic, antihypertensive, anti-asthmatic, antimicrobial, antiparasitic, antioxidant, and anticancer effects [36]. Some authors have examined the medicinal properties of *Nigella sativa* and suggested that it has anticancer and other health benefits [36,37,38]. Recently, DTQ was suggested to be useful for the management of COVID-19 infection [39,40].

CYP450 is the major oxidative enzyme responsible for the metabolism of a huge number of compounds in the human body. The number of predicted metabolites produced by the actions of different enzymes in DTQ is shown in Supplementary Figure S2. BioTransformer is a software tool that combines knowledge-based and machine learning approaches to predict the metabolisms of small molecules and aid metabolite identification [41,42].

The high binding energy for the complex obtained by docking indicated that the complex was stable [43]. Further, the RMSD graph provided supporting data regarding stabilization of the complex. H-bonds demonstrated strong interaction between the MSTN cavity residues and ligand DTQ. The average potential energy for DTQ–MSTN was found to be −2.311 KJ/mol. The temperature of the simulation system rapidly approached the target value (300 K) and remained stable throughout the simulation process, and the average temperature was 299.757 K. Pressure fluctuated widely and averaged 3.2 bar. The average density over 100 ps was 973.749 kg m^−3^. Density values were remarkably constant over time, indicating that pressure and density in the system were well balanced (Supplementary Figure S3).

In addition, the interaction between MSTN and ActR2B was checked using the Patch Dock server [44]; refinement and rescoring of docking solutions were performed using FireDock [45] to determine the global energy and different interacting amino acid residues. The global energy of the interaction between MSTN with ActR2B was −47.75, which agreed with a previous study [24]. PPI strategy employed to reveal the mechanism of MSTN to ActR2B binding. DTQ was able to reduce the number of interactions between MSTN and ActR2B in the complex. H-bonds and hydrophobic interactions between MSTN and ActR2B are shown in Figure 5. These bindings were found to be reduced by placing DTQ in the MSTN–ActR2B complex (Figure 6).

Currently, no MSTN inhibitors are available for medical use, though they are generally considered to be potential treatments for muscle-related diseases [46]. In the present study, binding free energies obtained by structure-based virtual screening showed that DTQ inhibits the activity of MSTN, by forming a complex with MSTN–ActR2B.

## 4. Materials and Methods

### 4.1. Natural Compounds Library Preparation 

A phytochemicals library composed of approximately 2000 compounds derived from different traditional medicinal plants was designed. These compounds were mainly taken from published studies on disease management and screened for potential anti-MSTN agents. Compounds were retrieved from the PubChem database for analysis.

### 4.2. Pharmacokinetics Properties of the Selected Compound

SwissADME was used to check the physicochemical parameters for drug design, including ADME. SwissADME is a web tool that provides free access to the physicochemical properties, pharmacokinetics, drug-likeness, and medicinal chemistry friendliness of the ligands. Different approaches (The BOILED-Egg, iLOGP, and Bioavailability Radar approaches) were used to verify the drug-likeness of the selected compounds [47,48]. Pred-hERG webserver was incorporated to check the toxicities of compounds and for rapid screening of compound libraries, in which green fragments indicate a contribution to hERG blockage, pink indicates a contribution to hERG blockage reduction, and gray indicates no contribution [49]. pkCSM was used to check ADMET properties for drug development. This is a freely accessible web server (http://biosig.unimelb.edu.au/pkcsm/prediction (accessed on 15 June 2021) that provides an integrated platform for pharmacokinetic and toxicity properties [50].

### 4.3. BioTransformer

BioTransformer is an open and freely accessible tool (www.biotransformer.ca (accessed on 16 June 2021) used to identify the metabolites and can generate the predicted structures of metabolites. Metabolism was predicted for phase one (CYP450) transformation by putting the Canonical SMILES of selected compounds in this tool [42].

### 4.4. Preparation of the Receptor Structure and Interaction Study

The protein data bank provided the crystal structure of MSTN (PDB ID: 3HH2) [45]. Water molecules and other atoms were removed, and Discovery Studio visualizer was used to prepare the 3D structure of the monomer for screening. Auto Dock Tool [51] was used to address the grid box at the active site of MSTN. The grid was generated with x, y, and z center values of −21.50, −13.61, and 28.70, respectively. The final ligand and protein structures were used for docking. Default values, such as number of GA run, were 10 with the population size (150), maximum number of evaluations (2,500,000), maximum number of generations (27,000), rate of gene mutation (0.02), and rate of crossing over (0.8), assuming movable ligand and rigid protein were used for docking. Docking results were visualized using Discovery Studio Visualizer. Further, ActR2B (PDB ID: 1S4Y) was used as a receptor for MSTN for the protein–protein interaction (PPI) study.

### 4.5. Protein–Protein Interaction Study

Freely available PatchDock servers were employed for PPI structural predictions [44]. PatchDock is a geometry-based molecular docking algorithm. PPIs between MSTN (with or without DTQ) and ActR2B were performed using default settings. The PatchDock algorithm is based on rigid docking. The results obtained were refined using Fire Dock, which is competent at refining and rescoring rigid-body docking results [52]. The best interaction energies of complexes were used for final analysis.

### 4.6. Molecular Dynamics Simulation

GROMACS 5.1.4 was used to perform the simulation study [53]. To produce the topology file of ligands, the ProDRG server was used [54]. The complex was immersed in a dodecahedron box of extended simple point charge (SPC) water molecules. The solvated system was neutralized by adding sodium ions to the simulation box. The entire system was composed of 881 atoms of target protein, one ligand, one Na+ counter ion, and 47,712 solvent atoms. In a cubic box of 377.187 nm^3^, the complex was solvated. For energy minimization, the steepest descent algorithm was used for 50,000 steps with a cut-off value of 1000 kJmol^−1^. Bond lengths were constrained using the LINCS algorithm. Equilibration phases were carried out for 100 ps at NVT (constant number of particles, volume, and temperature) and NPT (constant number of particles, pressure, and temperature). Temperature coupling was performed using a V-rescale, which is a modified Berendsen-thermostat, for immersion at 300 K with a time constant of 0.1 ps, and pressure coupling was completed with a Berendsen bath using a time constant of 2.0 ps [55]. MD simulation was conducted for 100 ns. Outcomes, that is, RMSD, RMSF, Rg, H-bond, SASA, temperature, pressure, density, and potential energy of the MSTN–DTQ complex, were analyzed according to the time-dependent behaviors of MD trajectories.

## 5. Conclusions

Due to the lack of natural potential inhibitors of MSTN for the management of SM-related disorders, we screened 2000 natural compounds by structure-based virtual screening, and we performed dynamic simulations for molecular interactions with MSTN to identify potential MSTN inhibitors. Finally, the four top compounds were selected based on their drug-likeness properties. Of these four compounds, DTQ had the better binding free energy (−7.40 kcal/mol). Consequently, DTQ was found to inhibit MSTN most potently and reduce binding between MSTN and ActR2B. We hope these findings aid the design of novel therapeutic MSTN inhibitors.

## Figures and Tables

**Table 1:** Physicochemical properties of selected compound.

Properties		Dithymoquinone (DTQ)	Calycosin	Limonin	Nigellidine
Physicochemical properties	Formula	C_20_H_24_O_4_	C_16_H_12_O_5_	C_26_H_30_O_8_	C_18_H_18_N_2_O_2_
	Molecular weight (g/mol)	328.40	284.26	470.51	294.35
	H-bond acceptors	4	5	8	2
	H-bond donors	0	2	0	1
	TPSA (Å^2^)	68.28	79.90	104.57	47.16 Å
Pharmacokinetics	GIA	high	High	High	High
	BBB	Yes	No	No	Yes
Drug-likeness	Lipinski Rule	Yes	Yes	Yes	Yes
	Ghose Rule	Yes	Yes	Yes	Yes
	Veber Rule	Yes	Yes	Yes	Yes
	Egan Rule	Yes	Yes	Yes	Yes
	Muegge Rule	Yes	Yes	Yes	Yes

**Table 2:** ADME properties of top selected compounds.

Property	Parameters	Dithymoquinone (DTQ)	Calycosin	Limonin	Nigellidine
Absorption	Water solubility (log mol/L)	−3.654	−3.423	−4.041	−3.651
	Caco2 permeability (log Papp in 10–6 cm/s)	1.367	0.96	0.922	1.304
	Intestinal absorption (human) (% Absorbed)	100	95.098	100	95.368
	Skin permeability (log Kp)	−3.189	−2.747	−2.832	−2.916
	P-glycoprotein substrate (Yes/No)	No	Yes	No	No
	P-glycoprotein I inhibitor (Yes/No)	Yes	No	Yes	Yes
	P-glycoprotein II inhibitor (Yes/No)	No	No	No	No
Distribution	VDss (human) (log L/kg)	−0.026	−0.326	0.265	0.508
	Fraction unbound (human) (Fu)	0.188	0.057	0.145	0.123
	BBB permeability (log BB)	−0.118	−0.315	−0.844	−0.104
	CNS permeability (log PS)	−2.719	−2.24	−3.07	−2.16
Metabolism	CYP2D6 substrate (Yes/No)	No	No	No	No
	CYP3A4 substrate (Yes/No)	Yes	Yes	Yes	Yes
	CYP1A2 inhibitor (Yes/No)	No	Yes	No	No
	CYP2C19 inhibitor (Yes/No)	No	Yes	No	Yes
	CYP2C9 inhibitor (Yes/No)	No	Yes	No	No
	CYP2D6 inhibitor (Yes/No)	No	No	No	No
	CYP3A4 inhibitor (Yes/No)	No	Yes	No	No
Excretion	Total clearance (log ml/min/kg)	−0.016	0.18	0.088	0.511
	Renal OCT2 substrate (Yes/No)	Yes	No	No	Yes
Toxicity	AMES toxicity (Yes/No)	Yes	Yes	No	No
	Max. tolerated dose (human) (log mg/kg/day)	0.534	0.141	−0.508	−0.425
	hERG I inhibitor (Yes/No)	No	No	No	No
	hERG II inhibitor (Yes/No)	No	No	No	No
	Oral Rat Acute Toxicity (LD50) (mol/kg)	1.649	2.127	3.452	2.423
	Oral Rat Chronic Toxicity (LOAEL) (log mg/kg_bw/day)	1.53	1.796	1.911	1.081
	Hepatotoxicity (Yes/No)	No	No	No	Yes
	Skin sensitization (Yes/No)	No	No	No	No
	*T. pyriformis* toxicity (log ug/L)	0.445	0.521	0.286	1.437
	Minnow toxicity (log mM)	1.323	0.397	0.446	1.17

**Table 3:** Estimation of binding energy obtained by multiple docking tools.

Target	Ligands Name	AutoDock Binding Energy (kcal/mol)	PyRx Binding Energy (kcal/mol)	Molecular Docking Server (kcal/mol)	SWISS Dock Binding Energy	
					ΔG (kcal/mol)	Full Fitness Score (kcal/mol)
MSTN	Dithymoquinone (DTQ)	−7.40	−6.60	−6.23	−6.47	−444.64
	Calycosin	−6.60	−6.88	−6.85	−6.65	−625.45
	Limonin	−6.85	−6.30	−6.35	−6.30	−643.54
	Nigellidine	−6.82	−6.65	−6.22	−6.45	−554.53

**Table 4:** List of number of H-bonds present in the DTQ–MSTN complex.

Target Name	Compound Name	H-Bond	H-Bond Distance (Å)
MSTN	Dithymoquinone (DTQ)	TYR38:OH-DTQ:O24	3.18
		TYR38:OH-DTQ:O23	3.18
		CYS43:N-DTQ:O19	3.21
		VAL103:N-DTQ:O2	3.1
		DTQ:O2-ALA40:O	2.81
		DTQ:O2-MET101:O	3.28
		DTQ:O3-CYS43:SG	3.69
		DTQ:O9-ASN41:O	3.12
		DTQ:O22-CYS43:O	2.86
		PRO76:CD-DTQ:O21	3.35

## Data Availability

Not applicable.

## Supplementary Materials

The following are available online. Figure S1: Graphical representation of the physicochemical properties of dithymoquinone (A) bioavailability radar (left), (B) BOILED-Egg analysis (middle), and (C) level of cardiotoxicity (right); Figure S2: Biotransformation of dithymoquinone into different metabolites as determined by phase one (CYP450) transformation. (A) Allylic hydroxylation, (B) Hydroxylation, (C) Hydroxylation of the terminal methyl, (D) Hydroxylation of the methyl carbon adjacent to the aliphatic ring of dithymoquinone, (E) Hydroxylation of the carbon alpha to the conjugated carbonyl of dithymoquinone, (F) Epoxidation of alkene, (G) Hydroxylation of the carbon gamma to the conjugated carbonyl, (H) Terminal desaturation, and (I) Alpha hydroxylation of the carbonyl group of dithymoquinone; Figure S3: Graphical representation of (A) potential energy, (B) pressure, (C) temperature, and (D) density of dithymoquinone-MSTN complex. Table S1: List of top 20 selected compounds with binding energy against myostatin obtained by AutoDock.

## Abbreviations

SM—Skeletal muscle, MSTN—Myostatin, DTQ—Dithymoquinone, ActR2B—Activin receptor type-2B, RMSD—Root-mean-square deviation, RMSF—Root-mean-square fluctuation, Rg—Radius of gyration, SASA—Solvent-accessible surface area, ADME—Absorption, distribution, metabolism, excretion, PPI—Protein–protein interaction.
