# Supplementary material for: Computational Identification of Dithymoquinone as a Potential Inhibitor of Myostatin and Regulator of Muscle Mass
Source: Molecules. 2021 Sep 6;26(17):5407. doi: 10.3390/molecules26175407 (PMC8434277; doi:10.3390/molecules26175407)
Supplement: Supplementary file 1 [file molecules-26-05407-s001.zip › molecules-1366847-supplementary.pdf]

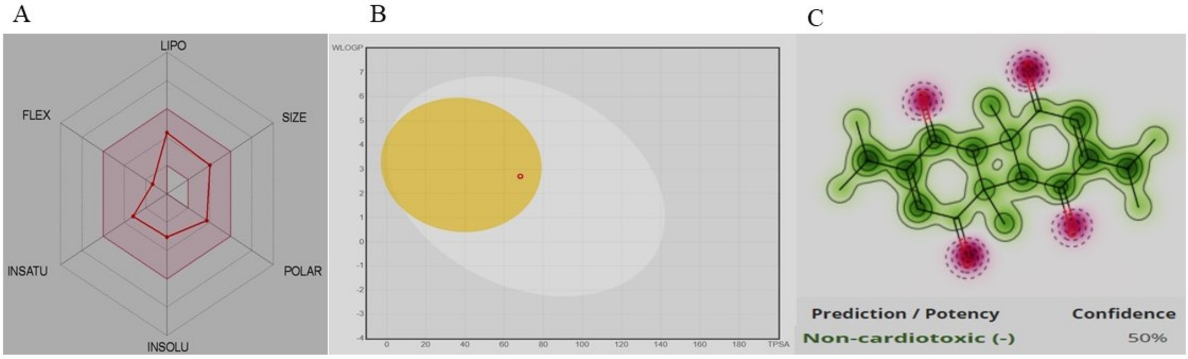

Figure S1. Graphical representation of the physicochemical properties of dithymoquinone **A)** bioavailability radar (left), **B)** BOILED-Egg analysis (middle), **C)** level of cardiotoxicity (right).

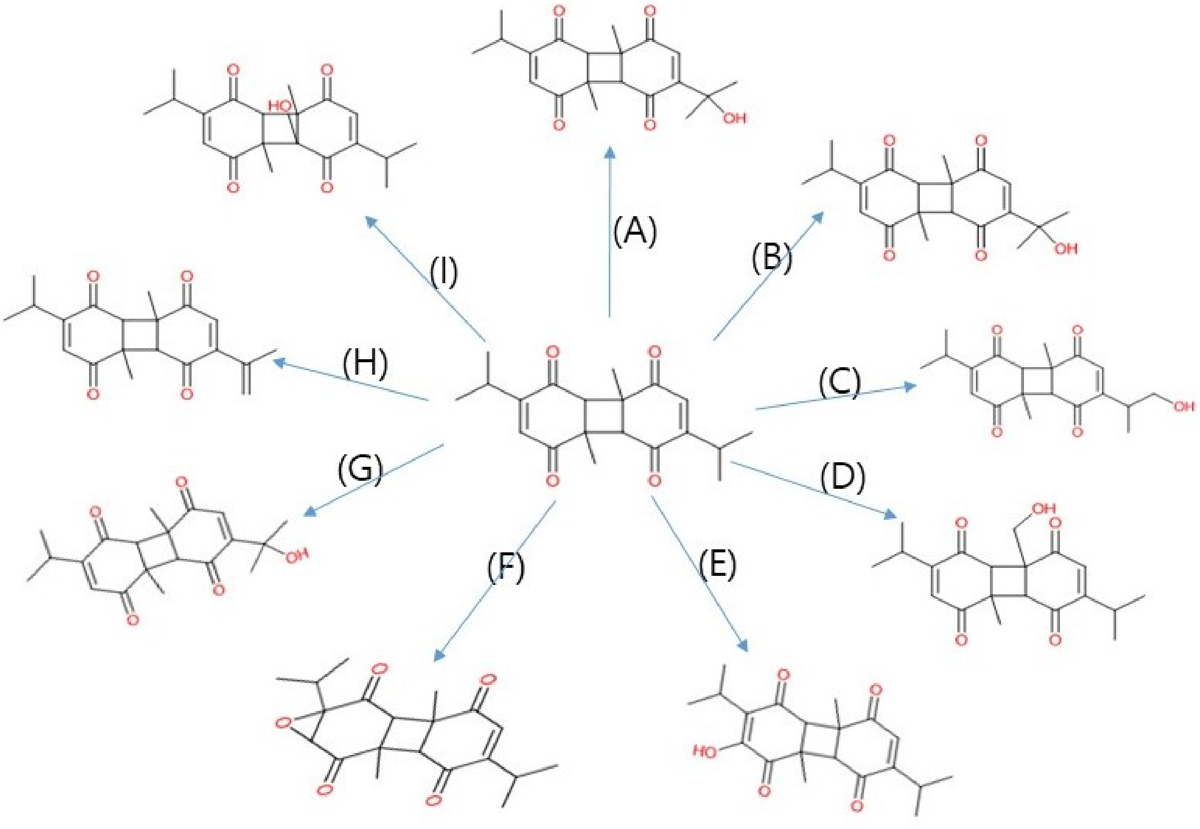

Figure S2. Biotransformation of dithymoquinone into different metabolites as determined by phase one (CYP450) transformation. A) Allylic hydroxylation, B) Hydroxylation, C) Hydroxylation of the terminal methyl, D) Hydroxylation of the methyl carbon adjacent to the aliphatic ring of dithymoquinone, E) Hydroxylation of the carbon alpha to the conjugated carbonyl of dithymoquinone, F) Epoxidation of alkene, G) Hydroxylation of the carbon gamma to the conjugated carbonyl, H) Terminal desaturation, I) Alpha hydroxylation of the carbonyl group of dithymoquinone.

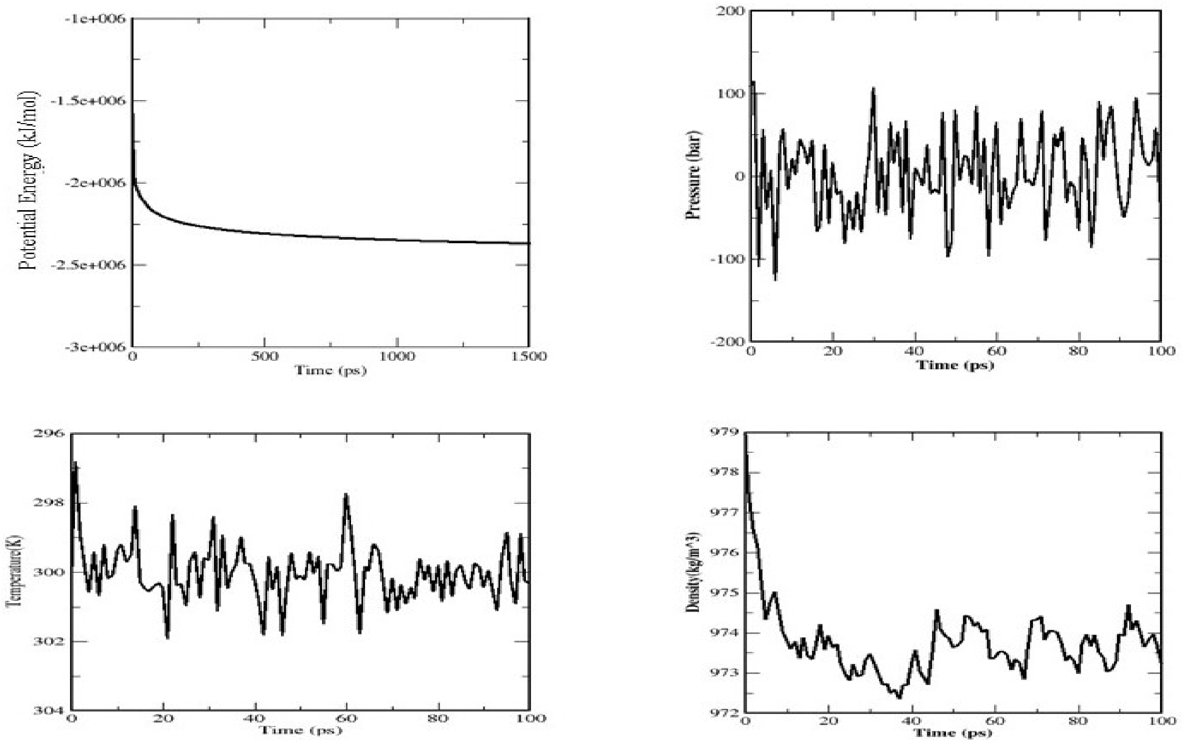

Figure S3. Graphical representation of **A)** potential energy, **B)** pressure, **C)** temperature, and **D)** density of dithymoquinone-MSTN complex.

Table S1. List of top 20 selected compounds with binding energy against myostatin obtained by AutoDock.

Compounds name	Binding energy (kcal/mol)	PubChem ID	Structure
Dithymoquinone	−7.40	398941	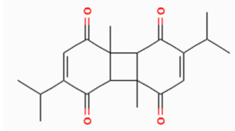
Calycosin	−6.60	5280448	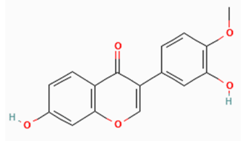
Limonin	−6.85	179651	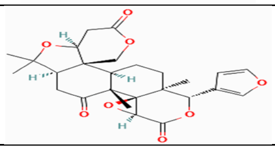
Nigellidine	−6.82	136828302	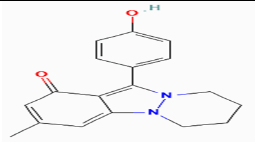
Thymoquinone	−5.21	10281	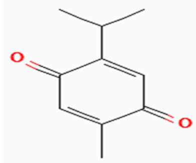
Galangin	−6.63	5281616	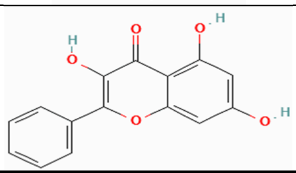

Carvacrol	−6.10	10364	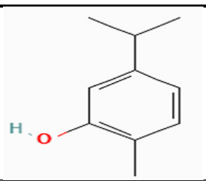
Kaempferol	−6.60	5280863	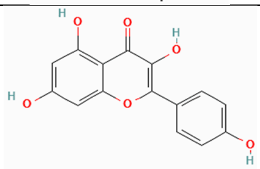
Thymol	−6.10	6989	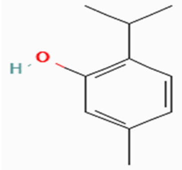
Carvone	−6.09	7439	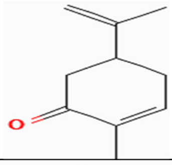
Limonenes	−6.21	405234158	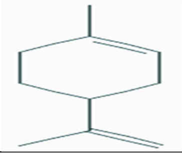
Citronellol	−6.01	8842	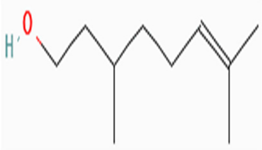
Thymohydroquinone	−6.68	95779	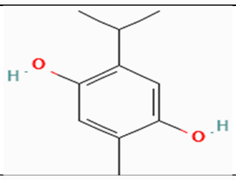
Nigellicine	−6.30	11402337	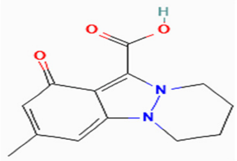
Nigellimine	−6.14	20725	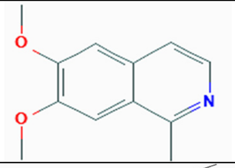
Chloroquine	−6.65	2719	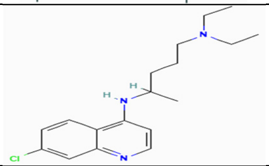
Hydroxychloroquine	−6.68	3652	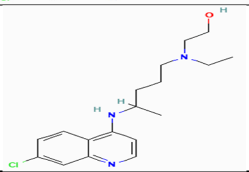

Coumarins	−6.35	54678486	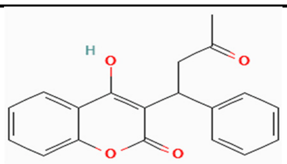
Stilbenes	−6.23	638088	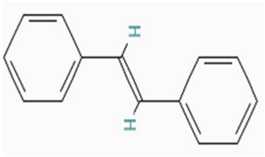
Sesamin	−6.31	72307	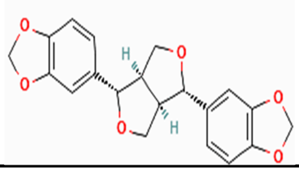
